# Exercise training is an effective alternative to estrogen supplementation for improving glucose homeostasis in ovariectomized rats

**DOI:** 10.14814/phy2.12617

**Published:** 2015-11-24

**Authors:** Tara L MacDonald, Kerry L Ritchie, Sarah Davies, Melissa J Hamilton, Daniel T Cervone, David J Dyck

**Affiliations:** Department of Human Health and Nutritional Sciences, University of GuelphGuelph, Ontario, Canada

**Keywords:** Estrogen, exercise, glucose tolerance, muscle, rats

## Abstract

The irreversible loss of estrogen (specifically 17-*β*-estradiol; E2) compromises whole-body glucose tolerance in women. Hormone replacement therapy (HRT) is frequently prescribed to treat estrogen deficiency, but has several deleterious side effects. Exercise has been proposed as an HRT substitute, however, their relative abilities to treat glucose intolerance are unknown. Thirty ovariectomized (OVX) and 20 SHAM (control) rats underwent glucose tolerance tests (GTT) 10 weeks post surgery. Area under the curve (AUC) for OVX rats was 60% greater than SHAM controls (*P* = 0.0005). Rats were then randomly assigned to the following treatment groups: SHAM sedentary (sed) or exercise (ex; 60 min, 5×/weeks), OVX sed, ex, or E2 (28 *μ*g/kg bw/day) for 4 weeks. OVX ex rats experienced a ∼45% improvement in AUC relative to OVX sed rats, whereas OVX E2 underwent a partial reduction (17%; *P* = 0.08). Maximal insulin-stimulated glucose uptake in soleus and EDL was not impaired in OVX rats, or augmented with exercise or E2. Akt phosphorylation did not differ in soleus, EDL, or liver of any group. However, OVX ex and OVX E2 experienced greater increases in p-Akt Ser473 in VAT and SQ tissues compared with SHAM and OVX sed groups. Mitochondrial markers CS, COXIV, and core1 were increased in soleus posttraining in OVX ex rats. The content of COXIV was reduced by 52% and 61% in SQ of OVX sed and E2 rats, compared to SHAM controls, but fully restored in OVX ex rats. In summary, exercise restores glucose tolerance in OVX rats more effectively than E2. This is not reflected by alterations in muscle maximal insulin response, but increased insulin signaling in adipose depots may underlie whole-body improvements.

## Introduction

Compared with age-matched males, premenopausal women display protection against insulin resistance (Nuutila et al. 1995; Donahue et al. 1997) attributed to the beneficial effects of estrogens on insulin action, glucose homeostasis, and body composition. The decline and eventual loss of circulating estrogen, specifically bioactive 17-*β*-estradiol (E2), is independently associated with increased risk for developing metabolic syndrome (reviewed by Carr 2003; Spangenburg et al. 2012b). Aside from a classical role in reproduction, estrogens confer a beneficial phenotype by regulating metabolic pathways, including mitochondrial function and biogenesis, fatty acid transport and oxidation, and lipogenesis and lipolysis (D’Eon et al. 2005; Rogers et al. 2009; Foryst-Ludwig and Kintscher 2010; Oosthuyse and Bosch 2012; Spangenburg et al. 2012a; Jackson et al. 2013; Velarde 2013). Rodent models of estrogen deficiency or ablated receptor signaling, such as estrogen receptor *α* (ER*α*) and aromatase knockout mice and ovariectomized (OVX) rats/mice, are similarly characterized by visceral adipose accumulation, reduced lipid oxidation, and increased ectopic accumulation of diacylglycerols (DAG) and ceramides, hyperinsulinemia, increased hepatic glucose output, and elevated inflammatory markers in adipose and liver. All of these perturbations converge to negatively influence whole-body glucose homeostasis (Jones et al. 2000; Misso et al. 2003; Bryzgalova et al. 2006; Ribas et al. 2010; Jackson et al. 2013).

Hormone replacement therapy (HRT) is primarily prescribed to treat symptoms of estrogen deficiency and curtail development of cardiovascular disease, osteoporosis, and cognitive impairments (Belchetz 1994; Keating et al. 1999). The ability of estrogens and HRT to regulate whole-body glucose homeostasis has gained considerable attention in women’s health research (Mauvais-Jarvis et al. 2013). Some formulas of HRT have favorable effects on glucose homeostasis (Andersson 1997; Kanaya et al. 2003; Margolis et al. 2004) while other studies have found the opposite and often attribute deteriorations in insulin sensitivity to the inclusion of progesterone with conjugated estrogens (Spencer et al. 2000). Regardless, the general efficacy and safety of HRT has been controversial since The Women’s Health Initiative (WHI) halted their large-scale HRT trials due to unacceptable hazard ratios for stroke, thrombosis, coronary heart disease, and elevated risk of endometrial, breast, and ovarian cancers (Rossouw et al. 2002). Despite the adverse health outcomes of HRT, it is undisputed that glucose metabolism is improved by estrogen-alone replacement in humans and rodents (reviewed in (Saengsirisuwan et al. 2009; Szmuilowicz et al. 2009). In order to circumvent the need for HRT, especially as the prevalence of T2D continues to increase worldwide, it is critical to develop alternative therapies/treatments that restore the positive metabolic effects of E2.

Since aerobic exercise is insulin-sensitizing (Wojtaszewski et al. 2000), training may be an effective substitute or adjunct for HRT (Spangenburg et al. 2012b). Evans et al. demonstrated comparable beneficial effects of exercise versus conjugated estrogen plus medroxyprogesterone in modulating glucose tolerance and body composition in healthy postmenopausal women (Evans et al. 2001). As evidenced in rodent studies, 12 weeks of exercise training initiated at the onset of OVX maintained normal skeletal muscle glucose uptake, prevented visceral adipose accretion, and improved whole-body glucose tolerance in OVX rats, indicating that exercise training averts metabolic dysfunction (Latour et al. 2001; Saengsirisuwan et al. 2009). However, to our knowledge, studies have not yet compared the abilities of exercise and E2 to treat, rather than prevent, glucose intolerance induced by estrogen deficiency.

The primary aim of the current study was to examine the effects of aerobic exercise training in treating impaired whole-body glucose homeostasis already present in OVX rats, and to compare this to an oral E2 treatment. Since skeletal muscle glucose uptake accounts for the majority of clearance from plasma, this was measured under basal and insulin-stimulated conditions in soleus and EDL. Insulin signaling and markers of mitochondrial content in soleus, EDL, liver, and adipose tissue were assessed to gain an understanding of tissue-specific effects of E2 depletion, and subsequent restoration or exercise training in OVX rats.

## Methods

### Materials and reagents

Reagents, molecular weight markers, and nitrocellulose membrane were purchased from BioRad (Mississauga, ON, Canada). Western Lightning Plus enhanced chemiluminescence (ECL) was purchased from PerkinElmer (NEL105001EA). The following primary antibodies were purchased from Cell Signaling: phospho-Akt Thr^308^ (cat. # 9275), phospho-Akt Ser^473^ (cat. # 9271), and Akt pan (cat. #4691). Antibodies against cytochrome oxidase complex IV (COXIV; cat. #16056), citrate synthase (CS; cat. #96600), and antiubiquinol-cytochrome C reductase core protein 1 (core1; cat. 110252) were acquired from Abcam. NP40 cell lysis buffer was acquired from Life Technologies and PMSF and protease inhibitor cocktail were obtained from Sigma (cat. #78830 and 9599). Insulin (Humulin, rDNA origin) was purchased from Eli Lilly (Toronto, ON, Canada).

### Animals

All procedures were approved by the Animal Care Committee at the University of Guelph and followed CCAC guidelines. Female Sprague Dawley rats were purchased from Charles River Laboratories at 4 months of age. Two days prior to arrival, all rats underwent either bilateral ovariectomy (OVX; 30 animals total) or SHAM surgery (SHAM; 20 animals total) under ketamine–atropine–xylazine anesthesia performed by Charles River technicians. Two flank incisions were made on the dorsal side, ovaries were identified, and were either removed by cauterization (OVX) or left intact (SHAM). OVX surgeries were verified during terminal experiments by recording uterine weight.

For the first 10 weeks, animals were grouped in cages of 3–4 as either SHAM or OVX with no further stratifications. Rats were housed in a temperature controlled room with a 12:12-h standard light–dark cycle. All animals had ad libitum access to food and were fed a phytoestrogen-purified, soy protein-free diet (Harlan 2020X, 16% calories from fat). A premeasured, excess amount of food was weighed and placed in each hopper and the amount leftover per cage was weighed and recorded every 2–3 days. Body mass was recorded weekly throughout the study.

### Glucose tolerance tests

At week 10, all rats underwent an intraperitoneal glucose tolerance test (ipGTT) to assess whole-body glucose homeostasis. Previous pilot work (T. MacDonald & D. J. Dyck, unpubl. data) determined that OVX rats were markedly glucose intolerant compared to SHAM controls at this time point. In order to allow comparisons between E2 and ex treatment approaches, versus prevention, confirmation of intolerance was required prior to administering either therapy. Rats were fasted for 6 h and injected with a 2 g/kg bolus of glucose. Blood glucose levels were measured from the tail vein at 0, 15, 30, 45, 60, 90, and 120 min using a handheld glucometer (FreeStyle Lite). Area under the curve (AUC) for the glucose response was calculated for each rat.

Tail vein blood samples were taken from each rat at 0 and 15 min in order to measure serum insulin. Samples were collected in microvette capillary tubes (Sarstedt Microvette 300 Z, Sarstedt, Nümbrecht, Germany), allowed to clot for 30 min and then centrifuged for 10 min at 10,000 *g*. The supernatant was removed and stored at −80°C for further analysis. At 15 weeks, GTTs and blood sampling procedures were repeated after a 4-weeks intervention period (outlined below).

After the 10-weeks GTT, SHAM rats were assigned to remain sedentary (SHAM sed) or undergo exercise treatment (SHAM ex). OVX animals were also allocated to sedentary (OVX sed) and exercise (OVX ex) groups, with an additional OVX group receiving oral 17-*β*-E2 treatment (OVX E2). All treatments were for 4 weeks. Rats were distributed among the various treatment groups such that each group had a similar average glucose tolerance, as determined by the mean AUC. This was done to prevent inherent differences in baseline glucose tolerance from skewing comparisons between groups after the 4-weeks treatments.

### Estrogen and exercise training protocols

#### Training protocol

Two days prior to the commencement of training, animals received two acclimatory training sessions (<5 min, 10 m/min, 0% incline). For the following 4 weeks, training took place on five consecutive days each week. Animals started at 10 m/min at a 0% incline for 20 min and the speed and incline were increased to 15 m/min at a 10% for 45 min by the fifth training session. Speed and duration were then increased to 16 m/min, with 5–10 min bursts of 20 m/min, totaling 60 min of total running time by the 10th training session. This was maintained for the remainder of the study. Since OVX rats had a visibly reduced running capacity, SHAM ex rats were matched daily to the training regime of the OVX group. This was done to ensure training effect comparisons were equal between SHAM and OVX groups.

#### Estrogen protocol

Rats assigned to E2 treatment received a daily dose of 17-*β*-estradiol delivered via chocolate hazelnut spread (Nutella), as described by Ingberg et al. (Ingberg et al. 2012). A concentrated stock solution of 17-B-E2 was made weekly by dissolving powdered estradiol in sesame oil to yield a concentration of 28 *μ*g 17-B-E2/5 *μ*L sesame oil/1 g nutella per kg body mass each day. All rats consumed the dose of estradiol in less than a minute. Rats not receiving estradiol received a similar dose of nutella with sesame oil as a control.

### Terminal surgeries and muscle glucose uptake experiments

Rats were allowed a 48-h recovery period following their last training session prior to surgical procedures in order to avoid the residual effects of the last bout of exercise. Animals were fasted overnight prior to terminal experiments and anesthetized with an intraperitoneal injection of pentobarbital sodium (6 mg per 100 g body mass) prior to all surgical procedures.

For glucose uptake, two strips from the soleus and EDL muscles of each animal were carefully dissected and assigned to one of the following conditions: (1) basal soleus; (2) insulin-stimulated soleus (10 mU/mL); (3) basal EDL; or (4) insulin-stimulated EDL (10 mU/mL). Glucose transport was calculated as accumulation of intracellular 3-*O*-[^3^H]methyl-d-glucose as reported previously (Mullen et al. 2007).

### In vivo *insulin signaling*

A third muscle strip from the soleus and EDL of one leg, as well as samples from perigonadal visceral adipose (VAT) and inguinal adipose (SC) from one side of the body, were removed and snap frozen as the basal condition for subsequent in vivo insulin signaling analysis. Animals were then injected with a 7.5 U/kg dose of insulin. Fifteen minutes later, samples from the contralateral leg (soleus and EDL), VAT, and SQ, as well as the liver were dissected, snap frozen, and stored as the insulin-stimulated condition for western blotting (Akt phosphorylation).

### Insulin ELISA

Serum samples from the 10- and 15-weeks GTTs were analyzed for insulin concentration using a commercially available kit (Millipore EZMRI-13K) according to manufacturers’ instructions. Accuracy and intra-assay comparisons were validated using two quality control standards (run in triplicate) provided with each kit. Serum samples were assayed in duplicates.

### Western blotting

Equal amounts (20 *μ*g) of basal and insulin-stimulated samples were separated by electrophoresis on 10% gels to assess content of phospho-Akt Ser^473^, phospho-Akt Thr^308^, and total Akt (Akt pan) in each tissue, following protocols published previously (Mullen et al. 2007; Stefanyk et al. 2011). Basal samples were separated on 10% or 15% gels to assess content of mitochondrial proteins in response to training (COXIV, CORE1, CS), GLUT4 and ponceau staining was used as a loading control. Bands were visualized using ECL and quantified using densitometry on Alpha Innovate Software.

### Statistics

All data are expressed as mean ± SE. For measures comparing mean values between SHAM sed, SHAM ex, OVX sed, OVX E2, and OVX ex basal, in the absence of any insulin or time points as factors, a one-way ANOVA was used to assess group differences. For measures involving insulin treatment or multiple time points, a two-way ANOVA was used. Tukey’s post hoc test was used in both ANOVA tests if significant differences were detected. In cases where data failed the Wilke–Shapiro test, a log(10) transformation was applied to normalize data. In all tables and figures, letters are used to denote statistical significance, such that means sharing a letter are not significantly different. All alpha values were set to *α* = 0.05.

## Results

### Ovariectomy induces hyperphagia and increased body mass

Throughout the duration of the study, OVX rats had significantly higher body mass relative to SHAM controls, regardless of sedentary, E2, or exercise treatments (Table1). Food intake was markedly increased in the OVX groups, but tapered over time (Table1) and was not significantly different from SHAMs by week 5. In pilot work, we tested the ability of pair-feeding OVX rats to SHAM controls to prevent changes in body mass induced by hyperphagia; however, this did not prevent greater weight gain post-OVX. Given this finding and the fact we did not want to restrict food intake in the OVX trained group, all groups were fed ad libitum.

**Table 1:** Body mass, food intake, and terminal uterine weight measurements

	1	2	3	4	5	6	7	8	9	10	11	12	13	14
Body weight (g)														
SHAM sed	218.0 ± 3.2a	257.4 ± 4.3a	282.6 ± 4.6a	300.1 ± 5.1a	316.2 ± 5.9a	329.3 ± 6.1a	340.7 ± 5.6a	346.9 ± 6.2a	349.3 ± 6.9a	343.3 ± 7.37a	359.1 ± 11.4a	363.8 ± 10.6a	364.8 ± 11.5a	373.2 ± 10.2a
SHAM ex											355.6 ± 11.2a	355.5 ± 11.6a	359.8 ± 10.0a	381.2 ± 9.9a
OVX sed	237.6 ± 2.4b	294.6 ± 2.9b	345.9 ± 3.9b	361.2 ± 4.4b	381.2 ± 4.5b	394.8 ± 5.2b	403.4 ± 5.8b	402.8 ± 7.2b	417.5 ± 6.0b	417.4 ± 6.0b	407.8 ± 11.9b	400.8 ± 12.0b	486.9 ± 12.1b	421.3 ± 12.9b
OVX E2											416.9 ± 9.7b	410.9 ± 9.9b	407.4 ± 9.5b	417.3 ± 9.9b
OVX ex											427.9 ± 11.7b	408.4 ± 10.7b	421.2 ± 9.6b	437.5 ± 12.8b
Food intake (kcal/day)														
SHAM sed	42.3 ± 2.8a	62.6 ± 2.1a	66.1 ± 1.7a	51.1 ± 1.9a	42.0 ± 1.3a	75.2 ± 2.9	69.1 ± 1.4	43.4 ± 1.4	58.5 ± 0.9	49.8 ± 0.5	53.0 ± 4.8ab	37.0 ± 3.9a	42.2 ± 2.3a	44.8 ± 2.0a
SHAM ex											60.9 ± 3.3a	39.9 ± 2.8ab	39.6 ± 1.3ac	40.1 ± 6.5ac
OVX sed	58.1 ± 1.7b	68.9 ± 0.7b	73.1 ± 0.8b	76.7 ± 3.3b	52.8 ± 1.4b	77.3 ± 1.8	68.5 ± 0.7	44.9 ± 1.8	59.6 ± 0.8	50.8 ± 0.4	51.7 ± 4.0ab	47.3 ± 1.5b	37.0 ± 0.6acd	36.6 ± 3.4bc
OVX E2											39.7 ± 2.5b	43.3 ± 1.8ab	34.9 ± 1.6bcd	35.1 ± 1.5bc
OVX ex											46.4 ± 1.3b	45.7 ± 0.9ab	33.6 ± 0.3bd	28.7 ± 1.4b
Uterine weight (g)														
SHAM sed														0.89 ± 0.09a
SHAM ex														0.95 ± 0.09a
OVX sed														0.16 ± 0.01b
OVX E2														0.28 ± 0.03b
OVX ex														0.13 ± 0.07b

Data are presented as mean ± standard error, *n* = 20 and 30 for SHAM and OVX rats from weeks 1 to 10, and *n* = 10 for all groups from weeks 11 to 14. Groups which share a letter within each parameter within each week are not statistically different, as assessed by unpaired *t*-test or one-way ANOVA. Statistical significance accepted at *P* < 0.05.

### Glucose tolerance is impaired in OVX rats at 10 weeks, is partially restored after 4 weeks of E2 treatment, and fully restored by exercise training

**Figure 1:**
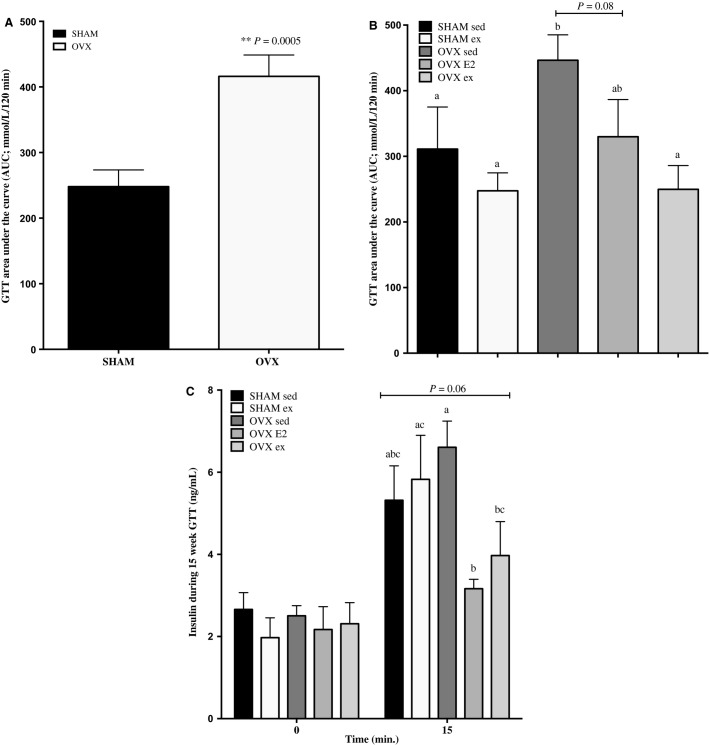
Calculated area under the curve (AUC) during (A) the 10-weeks GTT and (B) 15-weeks GTT. Plasma insulin concentrations at 0 and 15 min during the 15-weeks GTT are shown in (C). Data are presented as mean ± standard error, *n* = 20 and 30 for SHAM and OVX rats in panel (A), respectively, and *n* = 10 for all other groups. Groups which share a letter are not statistically different. Statistical significance accepted at *P* < 0.05. In (C), plasma insulin concentrations at the 15-min time point are trending toward statistical significance versus basal values, *P* = 0.06 as assessed by two-way ANOVA.

During the 10 weeks GTT, AUC was significantly increased in OVX rats relative to SHAM controls (Fig.1A). Glucose tolerance remained consistent in OVX sed rats at 15 weeks (416 ± 32, 10 weeks vs. 446 ± 39, 15 weeks; Fig.1B), whereas OVX E2 and OVX ex rats demonstrated 21% and 41% improvement (i.e. reduction) in their AUCs, respectively. Importantly, exercise restored GTT AUC to a greater extent than E2 compared to the OVX sed group.

### Plasma insulin response is not impaired in OVX rats during GTT

In order to assess whether reduced peak plasma insulin concentrations may have contributed to the whole-body glucose intolerance observed in OVX sed rats, or whether a greater initial plasma insulin response in OVX E2 and ex groups could explain the improved glucose tolerance, blood was sampled at 0 and 15 min during the GTT (Fig.1C). OVX sed rats had significantly elevated plasma insulin at 15 min (6.6 ± 0.6 ng/mL) relative to E2 or exercise-treated OVX groups (3.1 ± 0.2, 3.9 ± 0.8 pg/mL).

### Ex vivo glucose uptake in soleus and EDL is unchanged by OVX, E2, or exercise training

**Figure 2:**
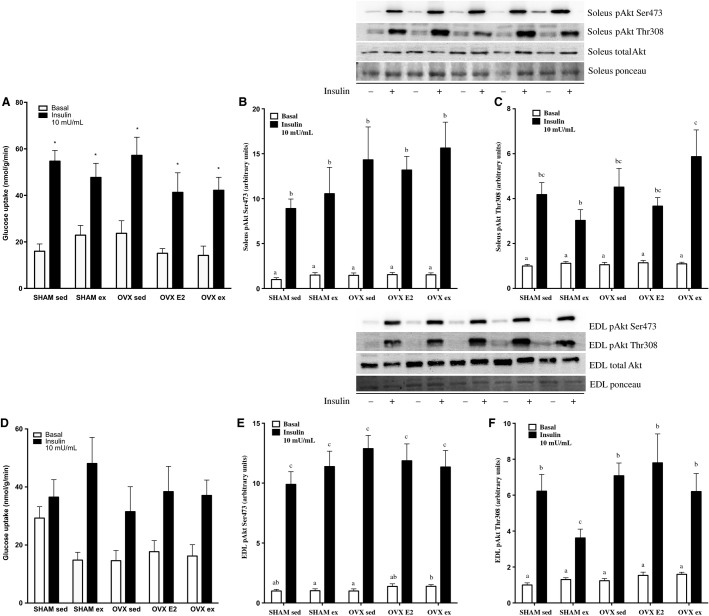
Basal and maximally insulin-stimulated glucose uptake in soleus and EDL (A, D), protein content of insulin signaling markers before (basal) and after (+insulin) 7.5 U/kg b.w injection in soleus p-Akt Ser 473 and p-Akt Thr308 (B, C) and EDL p-Akt Ser473 and p-Akt Thr308 (E, F). Data are expressed as mean ± standard error, *n* = 10 per group. Groups which share a letter are not significantly different. In (A), bars with an asterisk denote a significant insulin effect relative to the basal control within each group. Statistical significance is accepted at *P* < 0.05. In (D), *P* = 0.07 for the effect of insulin. In panel B, within +insulin group differences were as follows: OVX ex versus SHAM sed *P* = 0.06, OVX ex versus SHAM ex *P* = 0.08, OVX E2 versus SHAM sed *P* = 0.07, OVX ex versus SHAM ex *P* = 0.09; in panel C: OVX ex versus OVX E2 *P* = 0.09, SHAM ex versus SHAM sed *P* = 0.09 as assessed by two-way ANOVA.

Given that whole-body glucose disposal is primarily mediated by skeletal muscle (DeFronzo et al. 1981), we measured maximal insulin-stimulated 3-*O*-[^3^H]methyl-d-glucose uptake into soleus and EDL. There were no significant differences between SHAM or OVX phenotype or sedentary, E2, or exercise treatment in uptake in either muscle (Fig.2A and D). As expected, there was a significant effect of insulin in all groups.

### In vivo insulin signaling (pAkt) is improved in VAT and SC adipose tissue with E2 and exercise treatment in OVX rats

There were no differences in phosphorylation of either Akt Ser473 or Thr308 residues among the groups in soleus (Fig.2B and C) or in EDL (Fig.2E and F), which mirrored our glucose uptake data. p-Akt Thr308 was lower in the SHAM ex group of both tissues, total Akt was not different in basal or insulin-stimulated conditions in either soleus or EDL.

**Figure 3:**
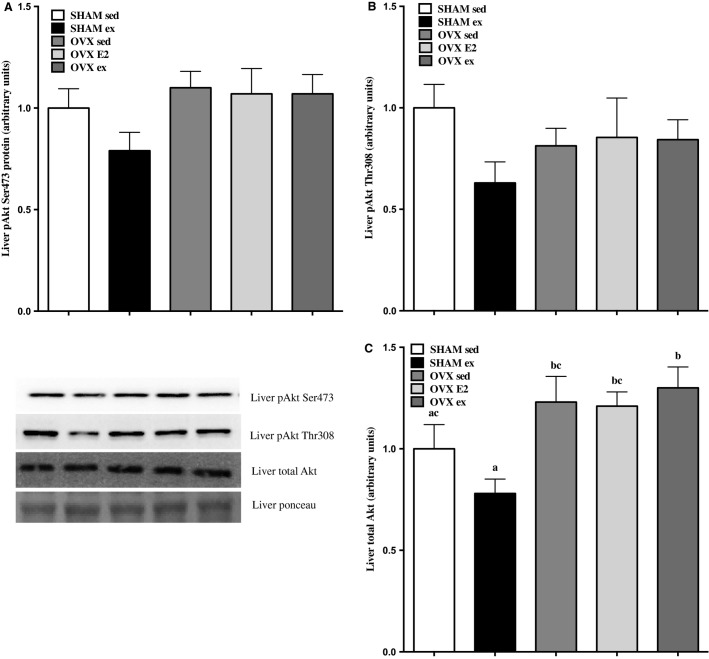
Protein content of insulin signaling markers before (basal) and after (+insulin) 7.5 U/kg b.w injection of insulin in liver (VAT) p-Akt Ser473 and Thr308 (A, B) and total Akt (C). Data are expressed as mean ± standard error, *n* = 10 per group. There were no statistical differences in content between groups on either Akt reside. Statistical significance is accepted at *P* < 0.05.

Changes in hepatic phosphorylation of p-Akt Ser473 (Fig.3A), p-Akt Thr308 (Fig.3B), and total Akt (Fig.3C) followed the same pattern, such that SHAM ex was reduced relative to all other groups (only statistically significant in total Akt). Therefore, the phosphorylation of Akt, at least under insulin-stimulated conditions, appears unaffected by OVX, E2, or ex.

**Figure 4:**
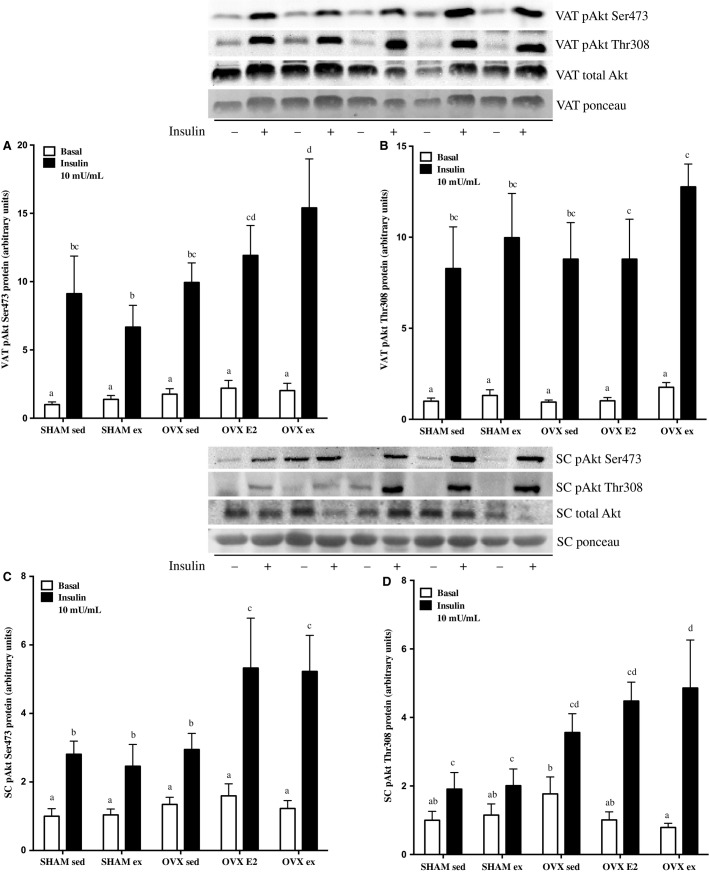
Protein content of insulin signaling markers before (basal) and after 7.5 U/kg b.w injection of insulin in visceral adipose tissue (VAT) p-Akt Ser473 and Thr308 (A, B) and subcutaneous adipose tissue (SC) p-Akt Ser473 and Thr308 (C, D). Data are expressed as mean ± standard error, *n* = 10 per group. Groups which share a letter are not significantly different. Statistical significance is accepted at *P* < 0.05. In panel B, within +insulin group differences were as follows: OVX ex versus OVX sed *P* = 0.08, OVX ex versus SHAM sed *P* = 0.053 as assessed by two-way ANOVA.

In VAT (Fig.4A and B), insulin-stimulated pAkt Ser473 was highest in OVX ex rats, and significantly greater than SHAM sed, SHAM ex, and OVX sed groups. Group differences were less robust with the Thr308 residue, but still reflected the same pattern, as OVX ex insulin-stimulated p-AktThr308 showed trends for differences from SHAM sed and OVX sed groups (*P* = 0.08 and 0.053, respectively; Fig.4B). Basal and insulin-stimulated p-Akt Ser and Thr were not reduced in VAT from OVX sed rats relative to SHAM sed controls, per se, but the relative fold increase was less than E2 or ex groups.

In SC adipose tissue (Fig.4C and D), OVX E2 and OVX ex rats showed significantly greater insulin-stimulated p-Akt Ser473 relative to SHAM and OVX sed groups (Fig.4C). This finding was mirrored by p-AktThr308 (Fig.4D), where OVX ex rats had significantly higher levels than SHAM sed or ex rats. Since there were statistical differences in basal p-Akt Thr308 in SC adipose, the fold-change between basal and insulin-stimulated phosphorylation in each group was also calculated to determine relative insulin induction of the Akt signaling cascade. OVX ex rats displayed the greatest increase (7.6 ± 2.5 fold), which was significantly greater than SHAM sed and ex rats, but not OVX sed or OVX E2 groups. The OVX E2 group exhibited a 4.2 ± 0.8 fold change, whereas SHAM sed, SHAM ex, and OVX sed only increased AktThr308 phosphorylation by 0.8 ± 0.3, 0.8 ± 0.4, and 1.7 ± 0.5 fold. Total Akt expression was not different under basal or insulin-stimulated conditions in VAT or SC tissues.

### Total GLUT4 content is not changed by OVX, E2, or exercise

**Figure 5:**
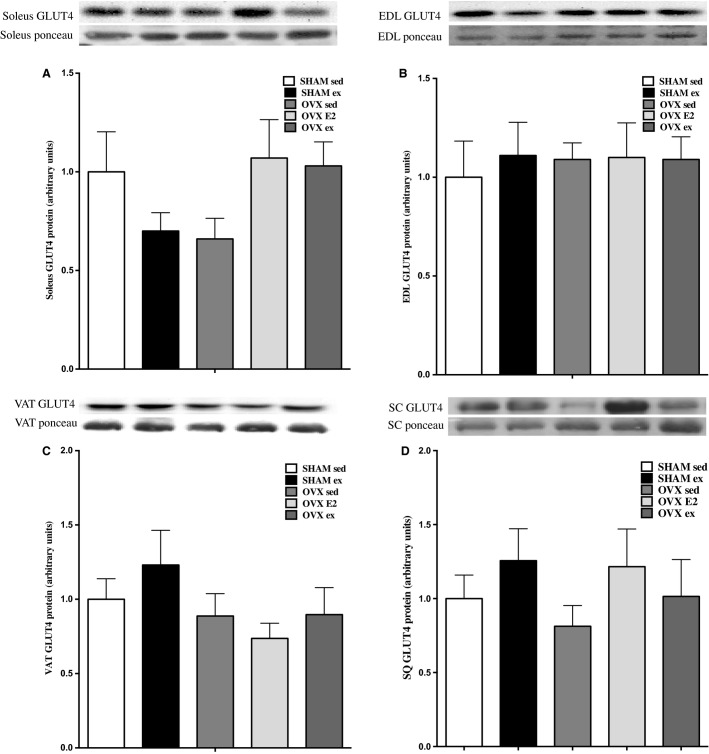
GLUT4 protein content in soleus, EDL, VAT, and SQ tissues (A–D). Data are expressed as mean ± standard error, *n* = 10 per group. Groups which share a letter are not significantly different, as assessed by one-way ANOVA. Statistical significance is accepted at *P* < 0.05.

Total tissue content of GLUT4 protein (quantified at the 45 kDa band) was not different in soleus, EDL, VAT, and SC adipose tissue in SHAM versus OVX or after sed, E2, or exercise treatments, as shown in Figures5A–D.

### Markers of mitochondrial content are significantly increased by exercise training in soleus and SC tissues

**Figure 6:**
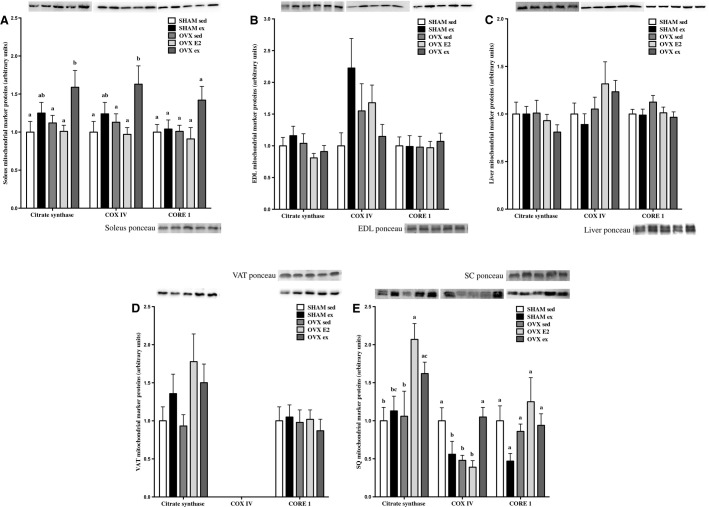
Protein content of mitochondrial markers citrate synthase, COXIV, and core1 in soleus, EDL, liver, VAT, and SQ tissues (A–E). Data are expressed as mean ± standard error, *n* = 10 per group. Within each target protein in each tissue, groups which share a letter are not significantly different. Statistical significance is accepted at *P* < 0.05. In panel A: between-group differences in core1 *P* = 0.06, in panel B: between-group differences in COXIV *P* = 0.06, in panel E: between-group differences in core1 *P* = 0.08 as assessed by one-way ANOVA.

Citrate synthase, COXIV, and core1 contents were measured to assess changes in markers of mitochondrial content after exercise or E2 treatments. Citrate synthase and COXIV were significantly increased in soleus after exercise training in OVX rats (Fig.6A), and core1 expression also approached significance (*P* = 0.06). Mitochondrial markers were unchanged by OVX, E2, or ex in EDL, liver, and VAT as shown in panels B, C, and D, respectively. In SC adipose tissue, both E2 and ex treatment increased citrate synthase in OVX rats to a greater extent than SHAM or OVX sed groups (Fig.6E). COXIV expression was significantly lower in SHAM ex, OVX sed, and OVX E2 groups, but robustly improved by exercise training in OVX rats. Changes in core1 approached significance in SC adipose tissue (*P* = 0.08), but overall content was not different between groups.

## Discussion

As women transition through perimenopause to irreversible estrogen deficiency, increased fat mass, particularly in the visceral depot, decreased lean body mass, and impaired whole-body glucose metabolism are commonly experienced (Szmuilowicz et al. 2009). Menopause induces a 60% increase in risk for developing metabolic syndrome independent of age, BMI, and physical activity (Park et al. 2003) and is often combated with various HRT regimes. The E2 dosage administered, as well as the inclusion of other hormones, namely progesterone, can elicit drastically different effects in vivo. Physiological levels of E2 can positively mediate glucose homeostasis, whereas high or low concentrations appear to be more detrimental (Muraki et al. 2006; Nagira et al. 2006). The dose administered in the present study was 28 *μ*g/kg bw/day, which has been shown in previous work to produce circulating E2 concentrations within a normal physiological range (Simpson 2003). Instead of implanting silasic capsules, which often cause an early E2 spike and subsequent taper in release (Ingberg et al. 2012), we adapted methods utilized by Isaksson et al. to elicit a physiological dosing regimen (Isaksson et al. 2011). Here, we chose to administer E2 in a sesame oil vehicle delivered within nutella in order to precisely manipulate the dose received per day and avoid large day-to-day variability. The palatability of nutella also ensured that the complete dose was ingested quickly. This methodology was effective in evoking a decline in hyperphagia, a common endpoint of E2 action.

Previous pilot work (T. MacDonald & D. J. Dyck, unpubl. data) and the current study demonstrate that body mass increases within 1 week post-OVX in rats. This persists with sedentary treatment and develops into profound glucose intolerance by 10 weeks, which is partially reversed with 4 weeks of E2 administration. Others have demonstrated the abilities of E2 treatment and exercise, independently and synergistically, to effectively attenuate metabolic dysfunction that occurs in estrogen-deficient women and OVX rodents, prior to the onset of disrupted glucose homeostasis. Preventative effects include lowered fasting plasma glucose, improved insulin response during a GTT, and reduced fat mass in humans (Evans et al. 2001) and rodents (Saengsirisuwan et al. 2009) after 8–12 weeks of exercise training or E2 administration, as well as improved insulin-stimulated skeletal muscle glucose uptake in OVX rats (Saengsirisuwan et al. 2009). The most novel finding in our current study is the ability of exercise to effectively reverse OVX-induced glucose intolerance from a treatment, as opposed to a prevention approach, whereas E2 was only partially restorative. After showing marked whole-body improvements with exercise and E2 treatments, we sought to investigate skeletal muscle function and cell signaling changes to gain an appreciation for how both treatments modulate glucose homeostasis.

Given that skeletal muscle accounts for ∼85% of glucose disposal (DeFronzo et al. 1981), we anticipated that muscle glucose uptake might explain the differences observed in the whole-body GTT responses. For this purpose, basal and insulin-stimulated uptake of 3-*O*-[^3^H]methyl-d-glucose uptake into isolated soleus and EDL was measured. Previously reported effects of OVX on ex vivo muscle glucose uptake have been equivocal. Saengsirisuwan et al. reported 29% and 43% reductions in maximal insulin-stimulated 2-deoxyglucose (2-DG) uptake in soleus and EDL of OVX rats (Saengsirisuwan et al. 2009). Impairments were prevented with E2 and exercise treatment independently, but not synergistically. Conversely, Gorres et al. found no impairment in skeletal muscle 2-DG uptake in OVX rats under similar experimental conditions, despite rats being sedentary or further challenged with a high-fat diet (Gorres et al. 2011). Here, we show no impairment in basal or maximal insulin-stimulated glucose uptake in soleus or EDL in the OVX sed group, and no augmentation after E2 or exercise treatment in either SHAM or OVX rats. Insulin-stimulated phosphorylation of Akt Thr308 and Ser473 residues mirrored the functional glucose uptake data in both muscles, such that there were no discernible effects of OVX, E2, or exercise treatment. Unfortunately, tissue yield was inadequate for measuring plasma membrane GLUT4 content, but total tissue homogenate GLUT4 remained unchanged in soleus and EDL. It is possible that while maximal insulin-stimulated glucose uptake and insulin signaling were unaffected, changes in sensitivity elicited by a half maximal dose may have occurred and gone undetected.

The discrepant finding of whole-body glucose intolerance in the absence of impaired, albeit maximal, insulin signaling and glucose uptake in soleus and EDL in OVX sed rats prompted us to measure markers of insulin signaling in other insulin-sensitive tissues. Interestingly, the increases in pAkt Ser473 and Thr308 content from basal to insulin-stimulated condition in VAT were highest in OVX ex rats versus SHAM and OVX sed/E2 groups. This was even more pronounced in the SC adipose depot, where 4 weeks of E2 or exercise treatment significantly increased insulin induction of Akt phosphorylation on the Ser473 site. Similarly, the fold increase in SC Akt Thr308 phosphorylation was significantly blunted in the OVX sed group, but recovered in E2 and exercise groups. Taken together, this would suggest that exercise training and E2 administration induced adipose tissue-specific effects that may have led to improvements in glucose tolerance. It is possible that exercise and E2-driven increases in Akt phosphorylation could increase VAT and SC adipose tissue glucose uptake leading to improvements in whole-body glucose tolerance. It is not known whether E2 or exercise can cause increases in glucose uptake in adipose tissue of an estrogen-deficient rodent. However, this could be important since OVX rats accumulate up to 70% more SC fat than SHAM controls, and there would be a greater absolute amount of tissue able to clear glucose during a GTT challenge (Zoth et al. 2012). Orava et al. recently demonstrated that glucose uptake into SC adipose tissue is similar to skeletal muscle in healthy subjects, on a per tissue mass basis, which suggests that SC adipose compartments may represent an important site for whole-body glucose homeostasis (Orava et al. 2011). Limitations to the current study are that neither body fat content nor glucose uptake or membrane-localized GLUT4 expression were measured in isolated fat depots to assess this possibility.

Perhaps the most encouraging information for justifying exercise as an alternative to synthetic E2 administration is the degree of metabolic benefit incurred from relatively short-term, medium intensity exercise. The treadmill training protocol was reduced in duration and intensity from previous studies used by our group (Smith et al. 2007; Ritchie et al. 2011) as the OVX females had visible limitations in exercise capacity. Nonetheless, this modified, less intense, training program still induced robust improvements in whole-body glucose tolerance in the OVX rats comparable to those seen with E2 administration. It is important to note that SHAM ex rats were exercise-matched to OVX ex rats, such that they completed the same duration and intensity (speed, incline). The SHAM ex groups would presumably have been exercising at a lower relative workload, and thus did not demonstrate the same degree of increased mitochondrial content as the OVX rats subjected to a relatively more intense exercise regime.

In OVX ex rats, markers of mitochondrial content were increased in soleus, but not EDL, likely indicating a lack of fast-twitch recruitment. In another study, by 8 weeks post-OVX rats demonstrated lower ATP synthesis, blunted citrate synthase activity, lower use of palmitoyl–carnitine and glycerol phosphate substrates, and decreased PGC-1a content, attributed to lower mitochondrial content in soleus and white gastrocnemius, all of which were reversible by E2 treatment (Cavalcanti-de-Albuquerque et al. 2014). Reductions in mitochondrial markers were minimal at 10 weeks post-OVX in the present study, and are consistent with previous findings of normal electron transport chain (ETC.) protein content in OVX mouse skeletal muscle (Jackson et al. 2013). However, subtle perturbations did occur in adipose tissue COXIV and core1 contents and interestingly, VAT and SC depots were most responsive to E2 and exercise-mediated increases in CS, COXIV, and core1. It is uncertain whether these are direct effects of E2 on VAT and SC tissue depots, or if changes in mitochondrial protein content are secondary to whole-body effects of E2 repletion, or whether increases in mitochondrial content are responsible for improvements in whole-body glucose tolerance.

## Conclusions

In conclusion, aerobic exercise training approximates the effects of E2 administration in reversing whole-body glucose intolerance observed 10 weeks post-OVX in female rats. The contributing impairments and subsequent improvements are not due to changes maximal insulin-stimulated glucose uptake in soleus or EDL muscle, or in the capacity of insulin to induce Akt phosphorylation in muscle or liver. Rather, the E2 and exercise-induced effects appear to be reflected in an adipose-specific manner, such that VAT and SC demonstrated the most robust increases in pAkt Thr308 and Ser473 content. Taken together, this study is the first to show that exercise is an effective therapy for restoring glucose tolerance in estrogen-deficient rats when compared with synthetic E2 administration, and suggests that exercise may provide an alternative option to HRT for estrogen-deficient women.

## Conflict of Interest

None declared.
